# Should the meniscal height be considered for preoperative sizing in meniscal transplantation?

**DOI:** 10.1007/s00167-017-4461-6

**Published:** 2017-02-23

**Authors:** Alfredo dos Santos Netto, Camila Cohen Kaleka, Mariana Kei Toma, Julio Cesar de Almeida e Silva, Ricardo de Paula Leite Cury, Patricia Maria de Moraes Barros Fucs, Nilson Roberto Severino

**Affiliations:** Irmandade da Santa Casa de Misericórdia de São Paulo, Rua: Dr. Cesário Motta Júnior 112, São Paulo, SP CEP 01221 020 Brazil

**Keywords:** Meniscus, Medial, Meniscus lateral, Tibial menisci, Joint, Knee, Transplantation, Graft, Accuracy, Dimensional measurement, Imaging, Magnetic resonance, MRI scans, Inter-observer variation

## Abstract

**Purpose and hypothesis:**

In preoperative sizing for meniscal transplantation, most authors take into consideration the length and width of the original meniscus, but not its height. This study aimed at evaluating (1) whether the meniscal height is associated with the meniscal length and width, (2) whether the heights of the meniscal segments are associated with the individual’s anthropometric data, (3) whether the heights of the meniscal segments are associated with each other in the same meniscus, and (4) the degree of symmetry of the meniscal dimensions between the right and left knees.

**Methods:**

In this cross-sectional, observational study, two independent radiologists measured the meniscal length, width and height in knee magnetic resonance imaging scans obtained from 25 patients with patello-femoral pain syndrome. Reproducibility of measurements was calculated with intraclass correlation coefficients. Associations between the anthropometric data and the meniscal measurements, the meniscal length and width versus height, and the heights of the meniscal segments in the same meniscus were examined with Pearson’s correlation.

**Results:**

Inter-observer reliability was excellent (>0.8) for length and height and good (0.6–0.8) for width measurements. There was also excellent agreement (>0.8) for the length and width of the menisci in the right and left knees. The heights of the horns of the lateral meniscus showed good agreement (0.6–0.8), while the heights of the other meniscal segments had excellent agreement between the sides (>0.8). There were significant associations with generally low (*r* < 0.5) correlation between the heights of the meniscal segments and the lengths and widths of the menisci, between the meniscal height and anthropometric data, and between the heights of the meniscal segments in the same meniscus. Correlations between anthropometric data and meniscal length and width were generally high (*r* > 0.7).

**Conclusions:**

There was excellent agreement between the meniscal dimensions of the right and left knees, and a weak association between the meniscal height with the meniscal width and length, between the height of the menisci with anthropometric data and between the heights of the segments in the same meniscus. The height of the meniscal segments may be a new variable in preoperative meniscal measurement.

## Purpose and hypothesis

The menisci have a fundamental role on the biomechanics of the knee, increasing the contact area between the femur and the tibia, transmitting and distributing the contact forces across a larger area of the articular cartilage and reducing the contact pressure on the cartilage. The absence of menisci increases the load across the surface of the articular cartilage and accelerates the occurrence of degenerative articular changes [1, 2].

Allograft meniscal transplantation is a therapeutic option for young and active patients who present with symptoms and limitations after total or subtotal meniscectomy [1–4]. The procedure restores the meniscal function in terms of load transmission, relieves symptoms and prevents the onset of degenerative changes while bringing back the normal mechanical contact across the articulation [5, 6].

In order to deliver an effective biomechanical functioning, the surfaces of the allograft meniscus must conform to those of the joint cartilage. The allograft meniscus must then be appropriately sized to the dimensions of the original meniscus to render a successful transplantation and promote optimal articulation congruency [2, 7–11].

In preoperative sizing for meniscal transplantation, most authors take into consideration the length and width of the original meniscus [11–16]. Calculations including the meniscal height have only been assessed in a few studies, despite the fact that the meniscus is a three-dimensional structure [8–10, 13]. Biomechanical studies have demonstrated that variations in the meniscal height result in significant changes in contact pressure on the articular surface [8, 10]. This indicates that the meniscal graft should have the same height as the native meniscus in order to properly distribute the load on the articular surface. A flatter meniscus, in contrast, may not provide such protection. We were unable to find in the literature studies assessing whether the meniscal height has any correlation with the meniscal length and width or with the individual’s anthropometric data. This knowledge may bring valuable information and improve the reliability of preoperative meniscal measurements, increasing the chances of success in meniscal transplantation.

The objectives of this study were to evaluate (1) whether the meniscal height is associated with the meniscal length and width, (2) whether the height of the meniscal segments is associated with the individual’s anthropometric data (weight and height), (3) whether the heights of the meniscal segments are associated with each other in the same meniscus and (4) the degree of symmetry of the meniscal dimensions between the right and left knees.

## Methods

This cross-sectional and observational study was performed in an outpatient clinic at a private university hospital. After approval of the study’s research project by the institution’s Ethics Committee for Research Involving Human Subjects (ECRIHS), we evaluated magnetic resonance imaging (MRI) scans of the knees of outpatients following up at the Knee Surgery Group at *Santa Casa de Misericórdia de São Paulo*. We included consecutive patients with patello-femoral pain syndrome who underwent MRI of both knees between September 2013 and June 2014. The exclusion criteria were the presence of skeletal immaturity, history of previous surgery on any one of the knees, any type of ligament or meniscal injury, or presence of tibio-femoral arthrosis. The cohort comprised 25 patients (50 knees) aged 18–41 years, including 13 men and 12 women. All participants signed an informed consent form before inclusion in the study.

All subjects underwent evaluation of weight (in kg) and height (in cm) by the same examiner. For weight measurement, the subjects were weighed on a mechanical scale that was calibrated before each measurement. The measurements were performed with the individuals barefoot and wearing light clothes, positioned upright at the centre of the scale, with their weight distributed on both feet. For height measurements, the individuals remained barefoot and upright, with their arms extended along their bodies and with their heads up against the stadiometer, along with their shoulders, buttocks and heels. The mobile part of the equipment was placed against the top of the individuals’ heads.

**Fig. 1:**
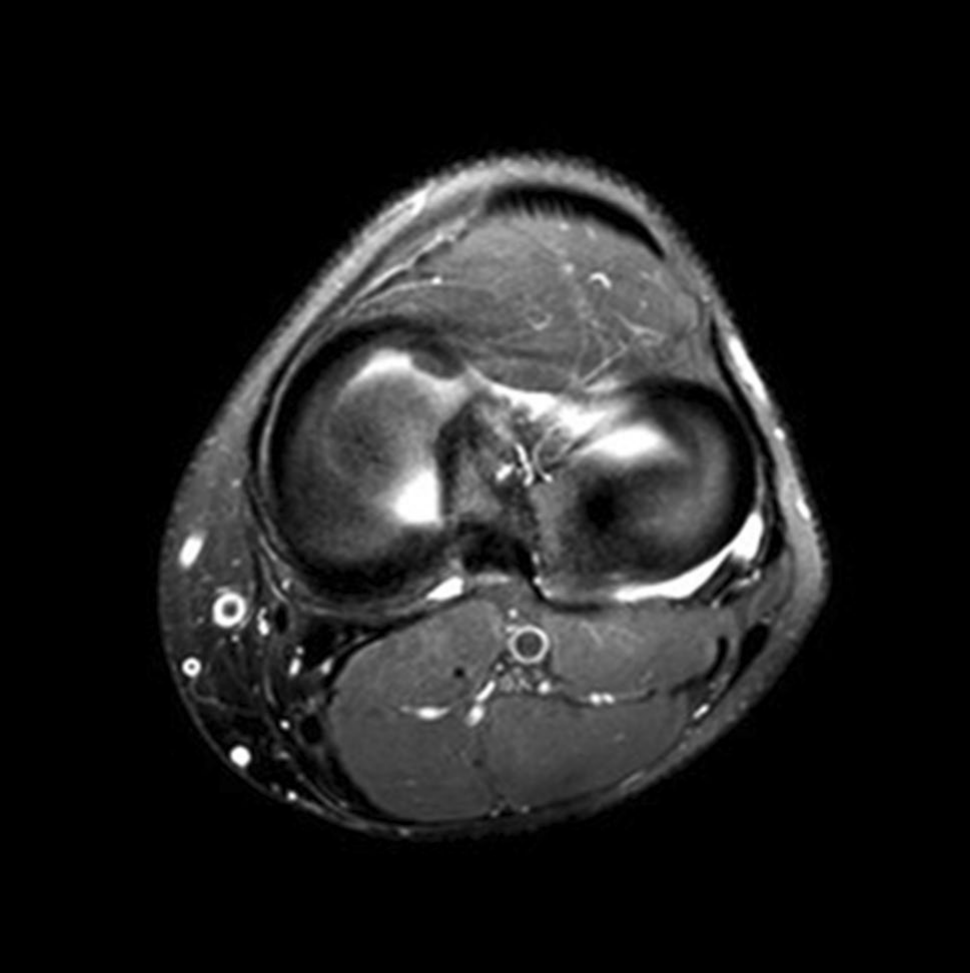
Proton-density-weighted sequences, acquired in the axial plane to demonstrate in a single image both menisci, the tibial insertion sites and the periphery of the menisci’ anterior horn, body and posterior horn

The MRI scans were obtained using a 1.5 T equipment (Intera, Philips) with a specific 8-channel coil and T1-, T2- and proton-density-weighted sequences in three planes (sagittal, coronal and axial). These sequences are used in all knee exams in our institution. We added only one proton-density-weighted sequence with thin slices, acquired in the axial plane (Fig. 1), directed to the tibio-femoral spaces (turbo spin echo with fat saturation, with the following parameters: repetition time 3393 ms and echo time 60 ms; matrix size (phase × frequency) 200 × 161; field of view 16 × 16 cm; slice thickness 1.0 mm with an interval of 0.3 mm, which allows the manipulation of the images with changes in plane and thickness).

The images were independently formatted in a workstation (Philips Extended Brilliance Workspace, v. 3.5.0.2250) by two radiologists experienced in musculoskeletal MRI, who manipulated the thickness and the orientation plane of the images to visualize the menisci in their longest axis in the axial plane, parallel to the tibial plateau, containing in the same image the tibial insertion site and the periphery of the meniscus’ anterior horn, body and posterior horn. Separate images were obtained for the medial and lateral menisci.

**Fig. 2:**
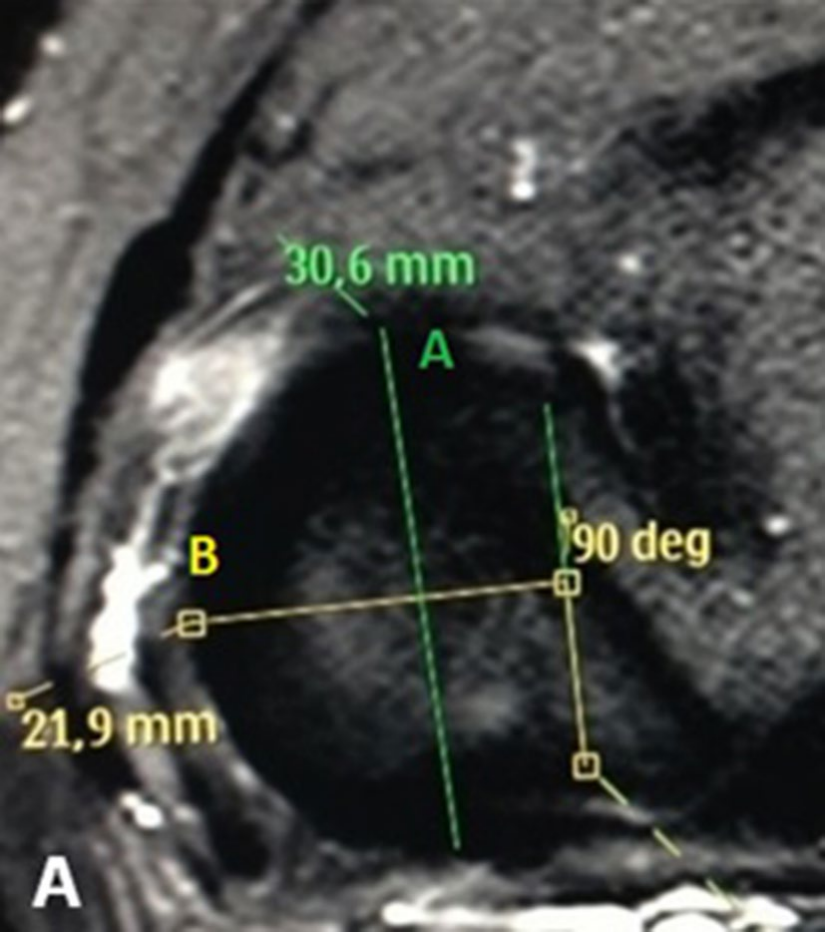
Measurement of the meniscal width and length in an axial slice. For the meniscal length, we identified the most anterior point of the anterior horn and the most posterior point of the posterior horn of the meniscus. A line traced between these points measured the meniscal length. In the picture below, the meniscal length is 30.6 mm (*green A line*). For the meniscal width, we traced a line between the most central points of the insertion sites of the anterior and posterior horns of the meniscus. In the midpoint of this line, we drew a perpendicular line up to the periphery of the outer contour of the body of the meniscus that was used to measure the meniscal width. In the picture below, the width is 21.9 mm (*yellow B line*)

We evaluated the images and determined the antero-posterior (length), medio-lateral (width) and longitudinal (height) measurements of the menisci. For the meniscal length, we measured the distance between the most anterior point of the tip of the anterior horn and the most posterior point of the tip of the posterior horn in an axial slice. To determine the meniscal width, we drew a line joining the most central points of the anterior and posterior horns’ insertion sites, and on the midpoint of this line, we drew a perpendicular line up to the periphery of the outer contour of the body of the meniscus. This line was also used to measure the width in the axial plane (Fig. 2).

**Fig. 3:**
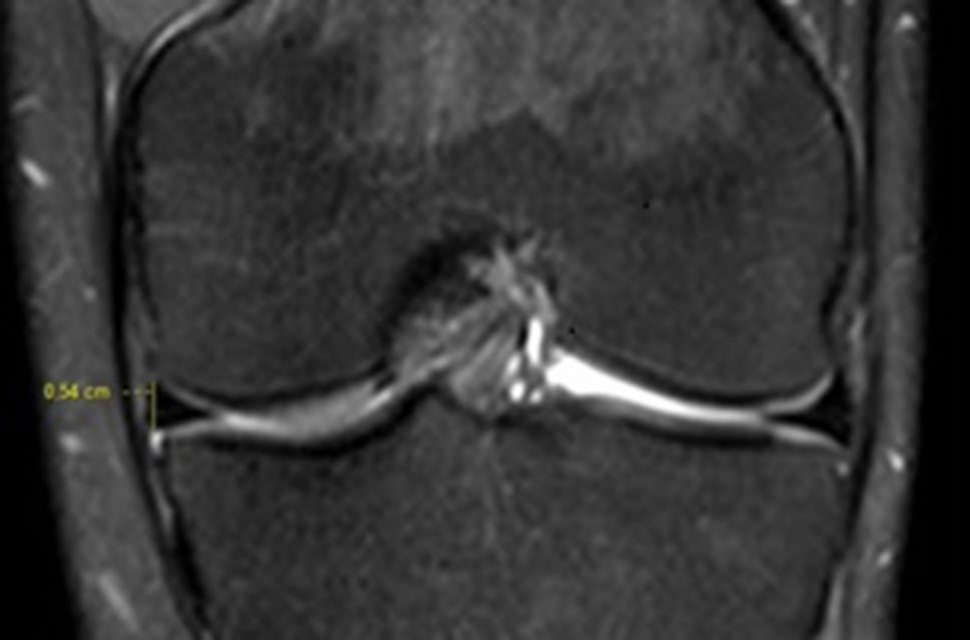
Height of the medial meniscal body, defined as the largest dimension in the longitudinal axis of the medial meniscus obtained in a coronal slice, at the same level in which the width of the medial meniscus was measured in an axial slice

To measure the meniscal heights, we performed a longitudinal measurement of each meniscal segment (anterior horn, body and posterior horn). The measurement of the height of the body was performed in a coronal slice, at the same level that the width of the meniscus was measured in the axial slice (Fig. 3).

**Fig. 4:**
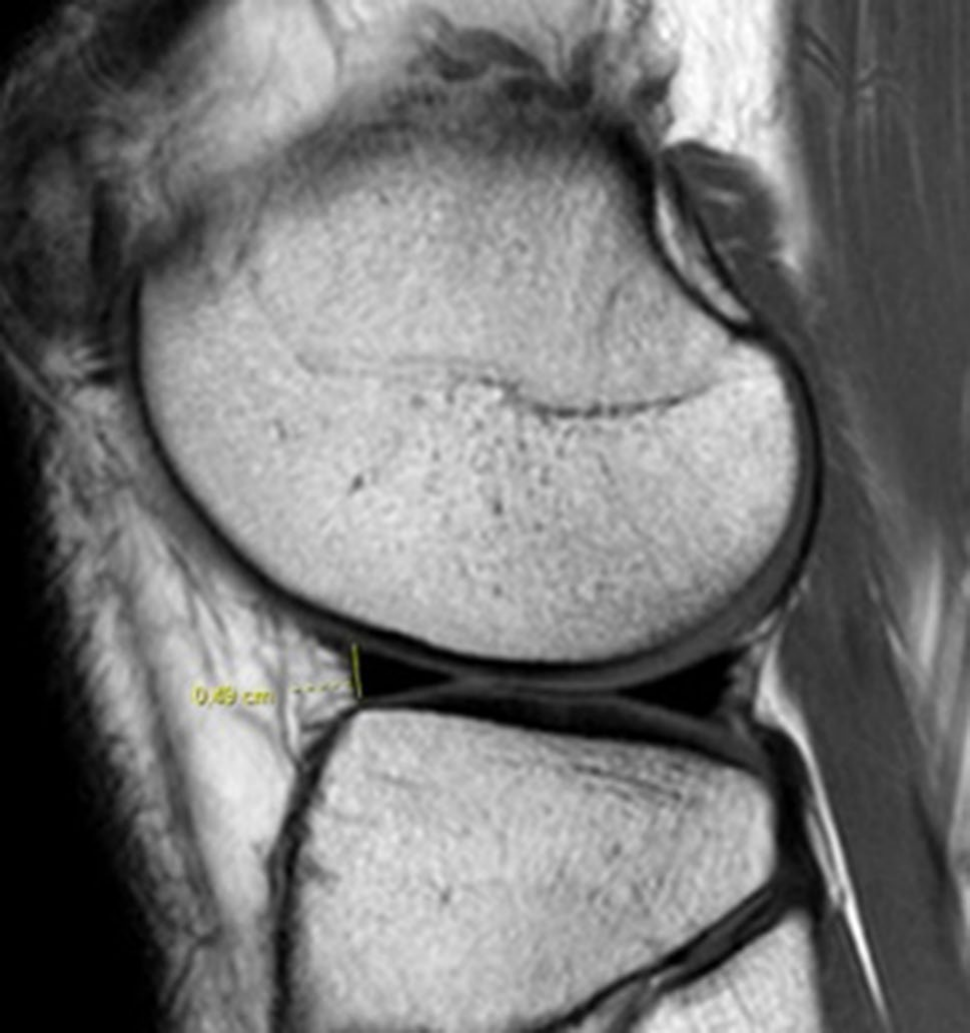
Height of the anterior horn of the lateral meniscus, defined as the largest dimension in the longitudinal axis of the anterior horn of the lateral meniscus obtained in a sagittal slice, at the same level in which the length of the lateral meniscus was measured in an axial view

The measurement of the heights of the anterior and posterior horns of each meniscus was performed in the sagittal slice at the same level that the meniscal length was measured in the axial slice (Fig. 4).

**Fig. 5:**
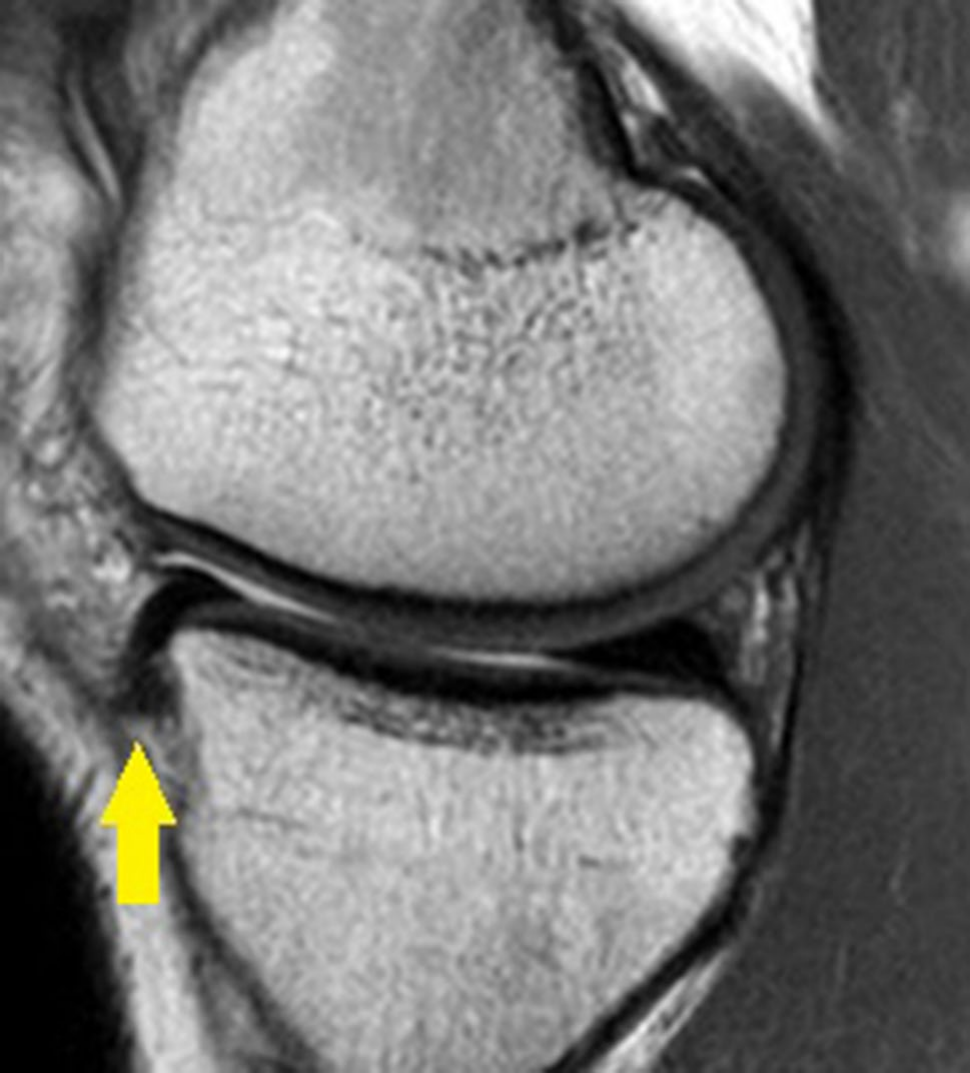
Anterior horn of the medial meniscus extending beyond the limits of the anterior tibial plateau margin (*arrow*)

We were unable to find a reproducible way to measure the height of the anterior horn of the medial meniscus because it extends beyond the anterior margin of the tibial plateau (Fig. 5). Hence, for the lateral meniscus we measured the heights of the three segments, and for the medial meniscus we only measured its body and posterior horn.

We organized the collected data in tables and analysed them with statistical tests. We used summary measures [mean and standard deviation (SD)] to describe the measurements obtained by each observer and calculated the intraclass correlation coefficients (ICCs) with their respective 95% confidence intervals (95% CIs) and repeatability measures to assess their reproducibility. We calculated the averages of the measurements obtained by both observers and described the measurements according to sides using mean and SDs. We also calculated the ICCs with their respective 95% CIs, and the repeatability measures to evaluate the agreement between the meniscal measurements obtained from the right and left sides.

We used Pearson’s correlations to analyse the association between the anthropometric data and the meniscal measurements, meniscal length and width versus height, and the heights of the meniscal segments in the same meniscus.

The ICC varies from 0 to 1, and the closer to 1 the greater the reproducibility (agreement) between the measurements.

We considered a significance level of 5% (*p* < 0.05).

## Results

Table 1 shows the mean and SD values of the meniscal dimensions. Inter-observer reliability was excellent (ICC >0.8) for length and height measurements and good (ICC between 0.6 and 0.8) for width measurements. There was also excellent agreement (ICC >0.8) for the length and width of the menisci in the right and left knees. As for the heights of the meniscal segments, the heights of the horns of the lateral meniscus, both anterior and posterior, showed good agreement (ICC between 0.6 and 0.8), while the heights of the other meniscal segments had excellent agreement between the sides (ICC >0.8) (Table 2).

**Table 1:** Measurements obtained by each observer and results of the reproducibility analysis

Measurement	Observer	Mean	SD	*N*	ICC	(95%) CI		Repeatability
						Inferior	Superior	
Medial body height	First	6.04	1.06	50	0.937	0.891	0.964	0.24
	Second	6.13	0.93	50				
Medial posterior horn height	First	6.06	1.04	50	0.957	0.926	0.976	0.21
	Second	6.08	0.93	50				
Lateral anterior horn height	First	4.83	0.71	50	0.943	0.902	0.967	0.17
	Second	4.86	0.73	50				
Lateral body height	First	6.57	1.05	50	0.955	0.921	0.975	0.21
	Second	6.48	0.97	50				
Lateral posterior horn height	First	5.81	0.76	50	0.893	0.817	0.938	0.24
	Second	5.90	0.73	50				
Medial length	First	45.33	3.39	50	0.889	0.809	0.936	1.22
	Second	44.85	4.05	50				
Medial width	First	32.88	3.02	50	0.622	0.420	0.766	1.91
	Second	32.17	3.30	50				
Lateral length	First	34.57	4.19	50	0.929	0.879	0.959	1.07
	Second	34.46	3.79	50				
Lateral width	First	31.99	3.52	50	0.711	0.542	0.824	2.08
	Second	31.57	4.20	50				

*SD* standard deviation, *N* number of knees, *ICC* intraclass correlation coefficient, *CI* confidence interval

**Table 2:** Measurements by sides and agreement between both sides

Measurement	Side	Mean	SD	*N*	ICC	(95%) CI		Repeatability
						Inferior	Superior	
Medial body height	Right	5.98	0.95	25	0.876	0.720	0.945	0.32
	Left	6.19	1.02	25				
Medial posterior horn height	Right	6.12	0.94	25	0.924	0.836	0.966	0.27
	Left	6.01	1.02	25				
Lateral anterior horn height	Right	4.89	0.68	25	0.777	0.559	0.895	0.34
	Left	4.80	0.75	25				
Lateral body height	Right	6.55	1.03	25	0.935	0.859	0.971	0.26
	Left	6.50	0.98	25				
Lateral posterior horn height	Right	5.95	0.73	25	0.681	0.403	0.845	0.40
	Left	5.76	0.73	25				
Medial length	Right	44.98	3.63	25	0.932	0.853	0.969	0.96
	Left	45.20	3.70	25				
Medial width	Right	32.31	2.55	25	0.835	0.665	0.924	1.15
	Left	32.74	3.17	25				
Lateral length	Right	34.82	3.91	25	0.915	0.809	0.962	1.09
	Left	34.21	3.99	25				
Lateral width	Right	31.44	3.43	25	0.918	0.791	0.966	0.94
	Left	32.13	3.76	25				

*SD* standard deviation, *N* number of knees, *ICC* intraclass correlation coefficient, *CI* confidence interval

There were statistically significant (*p* < 0.05) associations between the heights of the meniscal segments and the lengths and widths of the menisci, between the meniscal height and anthropometric data (weight and height), and between the meniscal segments’ heights in the same meniscus. Although these associations were statistically significant, the correlation values were generally low (*r* < 0.5) (Tables 3, 4, 5). We found statistically significant associations and generally high (*r*  > 0.7) correlations between anthropometric data and meniscal length and width (Tables 3, 4).

**Table 3:** Pearson’s correlation analysis of the heights of the medial meniscal segments with width, length and anthropometric data

Correlation	Medial length	Medial width	Weight	Height
Weight				
*r*	0.806	0.684		
*p*	<0.001	<0.001		
*N*	50	50		
Height				
*r*	0.719	0.648	0.744	
*p*	<0.001	<0.001	<0.001	
*N*	50	50	50	
Medial body height				
*r*	0.447	0.336	0.356	0.162
*p*	0.001	0.017	0.011	0.260
*N*	50	50	50	50
Medial posterior horn height				
*r*	0.454	0.356	0.414	0.350
*p*	0.001	0.011	0.003	0.013
*N*	50	50	50	50

*r* Pearson’s coefficient, *N* number of knees

**Table 4:** Pearson’s correlation analyses of the heights of the lateral meniscal segments with width, length and anthropometric data

Correlation	Lateral length	Lateral width	Weight	Height
Weight				
*r*	0.743	0.567		
*p*	<0.001	<0.001		
*N*	50	50		
Height				
*r*	0.711	0.738	0.744	
*p*	<0.001	<0.001	<0.001	
*N*	50	50	50	
Lateral anterior horn height				
*r*	0.398	0.080	0.436	0.210
*p*	0.004	0.583	0.002	0.143
*N*	50	50	50	50
Lateral body height				
*r*	0.441	0.322	0.506	0.467
*p*	0.001	0.023	<0.001	0.001
*N*	50	50	50	50
Lateral posterior horn height				
*r*	0.332	−0.015	0.302	0.179
*p*	0.018	0.917	0.033	0.214
*N*	50	50	50	50

*r* Pearson’s coefficient, *N* number of knees

**Table 5:** Results of Pearson’s correlation analyses of the heights of the meniscal segments

Correlation	Medial body height	Medial posterior horn height	Lateral anterior horn height	Lateral body height
Medial posterior horn height				
*r*	0.614			
*p*	<0.001			
*N*	50			
Lateral anterior horn height				
*r*	0.144	0.343		
*p*	0.319	0.015		
*N*	50	50		
Lateral body height				
*r*	0.445	0.479	0.417	
*p*	0.001	<0.001	0.003	
*N*	50	50	50	
Lateral posterior horn height				
*r*	0.082	0.341	0.574	0.473
*p*	0.570	0.015	<0.001	0.001
*N*	50	50	50	50

*r* Pearson’s coefficient, *N* number of knees

## Discussion

The occurrence of pain and repeated joint effusion after meniscectomy, especially in young and active patients, is a challenging problem for orthopaedic surgeons [6]. The therapeutic options for this group of patients include allograft meniscal transplantation and implantation of synthetic meniscal substitutes, which are currently under evaluation in different study phases. Future trends are meniscal substitutes developed with tissue engineering and able to mimic the complex biomechanical function of the menisci [17].

Human meniscal transplantation is no longer considered an experimental treatment since many clinical studies with hundreds of cases have been published on this topic in the international literature [6, 14, 18–22] and several animal and basic science studies have been conducted [23–25]. Although most studies have shown good short- and medium-term results, long-term evidence shows that the procedure is not curative, and progression of osteoarthrosis is observed. Patients then must be warned about the possibility of requiring in the future another surgical procedure in the knee [26, 27].

The margin of error between the sizes of the original meniscus and the graft must be as low as possible to increase the chances of success of the transplantation [7–10]. In a biomechanical study published by Dienst et al., the margin of acceptable error to maintain contact pressures in the articulation after meniscal allograft transplantation is 10% [7].

Several methods are available to evaluate the meniscal dimensions, including indirect methods using plain X-ray, computed tomography, MRI of the affected knee and anthropometric data, and direct methods using MRI of the contralateral, non-injured knee [15, 28–32]. Still, there is no consensus regarding the most reliable method to determine the ideal graft size [11–13, 15, 33].

Some authors defend that direct measurements of the meniscal size with MRI are more accurate than indirect measurements with plain X-ray or computed tomography [9, 31]. MRI of the contralateral, non-injured side is an alternative to direct measurement of the meniscal dimensions before transplantation. For such, it is necessary to prove that the human menisci are bilaterally symmetrical. Some authors have studied the symmetry between the human menisci and concluded that they are highly correlated [31, 34, 35]. In our study, we found similar results to those in the literature. The lengths and widths of both menisci and the heights of the medial meniscus showed good agreement between the right and left knees, while the heights of the lateral meniscus had fair agreement. Since we found a generally excellent agreement between the sides, and considering that MRI allows a three-dimensional meniscal measurement including measurement of the height of the meniscal segments, we believe that the direct measurement of the contralateral meniscus with MRI allows a more appropriate analysis of the dimensions of the ideal meniscal graft before homologous transplantation.

Some authors have correlated the meniscal dimensions with the patients’ anthropometric data and demonstrated that height, weight and gender have a direct correlation with the meniscal size [32, 36]. In our study, we found a statistically significant association between the weight and height of the studied individuals and the meniscal dimensions. Similar to the study by Stone et al. [36], we also found strong correlations between the anthropometric data and the length and width of the menisci (*r* > 0.7). In our study, we also evaluated the correlations between the anthropometric measurements and the meniscal height and found that the correlation values were generally low (*r* < 0.5).

Most authors base the measurement of the menisci on its length and width before allograft transplantation. It is fundamental to measure the meniscal width and length independently since the measurement of one-dimension cannot accurately predict the other [16]. In our study, we measured the length, width and height of the meniscal segments using MRI. We measured the meniscal length and width directly on the MRI image using reference lines drawn in the axial plane. Tissue banks usually measure the meniscal width as the distance from the peak of the intercondylar eminence to the periphery of the tibial plateau rather than using the lines in the axial plane. We believe that a direct measurement of the meniscal tissue is a better method than the use of bone landmarks, but in order to make these measurements, we manipulated the thickness and the orientation plane of the images in the axial plane of the MRI images, as described before.

There are few studies on meniscal transplantation evaluating the meniscal height, and we were unable to find any study taking into consideration the height of the meniscal segments. However, biomechanical studies have demonstrated the importance of the meniscal height in contact biomechanics in the articular surface [8, 10]. In a biomechanical study, Haut et al. demonstrated that variations greater than 0.5 mm in the medial meniscus height and larger than 1 mm in the lateral meniscus height resulted in significant changes in contact pressure on the articular surface [8].

We were also unable to find in the literature studies correlating the height of the meniscus with its length and width or with anthropometric data, or whether the heights of the meniscal segments are associated with each other in the same meniscus. Although our study found statistically significant associations between the meniscal height and different variables, the values of the correlations were generally low (*r* < 0.5), indicating that the correlations involving the height of the meniscal segments are poor. This was the most important finding of our study, as it brings new information about the meniscal dimensions. Since the meniscal length and width have a poor correlation with the meniscal height, we conclude that the meniscal height is an independent measure that should not be predicted from the meniscal length and width. Based on our findings and those from biomechanical studies indicating the importance of the meniscal height for the biomechanical functions of the meniscus on load protection, we believe that the height of the meniscal segments should be a new variable in preoperative meniscal measurement. This information may change the way the menisci are routinely measured before transplantation, in order to increase the reliability of the meniscal measurement and the success rates of meniscal allograft transplantation. This hypothesis should be confirmed in clinical studies to determine whether the preoperative evaluation of the meniscal height is able to change the clinical outcome after meniscal transplantation.

The limitations of our study were the relatively small number of cases and lack of comparison of the meniscal measurements obtained with MRI with anatomical measurements obtained with cadaveric dissection to confirm the accuracy of the measurements.

## Conclusions

We found excellent agreement between the meniscal dimensions of the right and left knees, and a weak association between the meniscal height with the meniscal width and length, between the height of the menisci with anthropometric data and between the heights of the segments in the same meniscus.

## Abbreviations

MRI: Magnetic resonance imaging
SD: Standard deviation
ICC: Intraclass correlation coefficients
